# Regulatory Network Structure as a Dominant Determinant of Transcription Factor Evolutionary Rate

**DOI:** 10.1371/journal.pcbi.1002734

**Published:** 2012-10-18

**Authors:** Jasmin Coulombe-Huntington, Yu Xia

**Affiliations:** 1 Bioinformatics Program, Boston University, Boston, Massachusetts, United States of America; 2 Department of Chemistry, Boston University, Boston, Massachusetts, United States of America; 3 Department of Biomedical Engineering, Boston University, Boston, Massachusetts, United States of America; The Centre for Research and Technology, Hellas, Greece

## Abstract

The evolution of transcriptional regulatory networks has thus far mostly been studied at the level of *cis*-regulatory elements. To gain a complete understanding of regulatory network evolution we must also study the evolutionary role of *trans*-factors, such as transcription factors (TFs). Here, we systematically assess genomic and network-level determinants of TF evolutionary rate in yeast, and how they compare to those of generic proteins, while carefully controlling for differences of the TF protein set, such as expression level. We found significantly distinct trends relating TF evolutionary rate to mRNA expression level, codon adaptation index, the evolutionary rate of physical interaction partners, and, confirming previous reports, to protein-protein interaction degree and regulatory in-degree. We discovered that for TFs, the dominant determinants of evolutionary rate lie in the structure of the regulatory network, such as the median evolutionary rate of target genes and the fraction of species-specific target genes. Decomposing the regulatory network by edge sign, we found that this modular evolution of TFs and their targets is limited to activating regulatory relationships. We show that fast evolving TFs tend to regulate other TFs and niche-specific processes and that their targets show larger evolutionary expression changes than targets of other TFs. We also show that the positive trend relating TF regulatory in-degree and evolutionary rate is likely related to the species-specificity of the transcriptional regulation modules. Finally, we discuss likely causes for TFs' different evolutionary relationship to the physical interaction network, such as the prevalence of transient interactions in the TF subnetwork. This work suggests that positive and negative regulatory networks follow very different evolutionary rules, and that transcription factor evolution is best understood at a network- or systems-level.

## Author Summary

Transcription factors (TFs) are proteins which regulate the expression of genes by interacting with DNA. Mutations in TF protein sequences can affect the expression levels of regulated genes throughout evolution. In this study, we look into the factors which cause the different TFs in baker's yeast to be more or less tolerant of mutations during recent evolution. This tolerance is measured as the evolutionary rate, defined for each protein as the relative rate of protein-changing DNA mutations over other mutations (K_a_/K_s_). We found that the typical determinants of protein evolutionary rate, such as expression level and network interactions have a very different influence on TF evolutionary rate. We found that TF evolutionary rate is most highly correlated to the evolutionary properties of the genes which they regulate and specifically genes which they activate. We also show that TF evolutionary rate predicts actual evolutionary expression differences of regulated genes and we discuss some of the features unique to TFs which likely contribute to their different evolutionary trends, such as the types of protein-protein interactions prevalent in the TF subnetwork or TFs' potential role in adaptive evolution.

## Introduction

The study of regulatory network evolution has so far mostly concentrated on *cis*-regulatory variation, such as the loss or gain of transcription factor (TF) binding sites in the promoter region of a gene. But *trans*-level variations are known to account for a significant amount of the expression variation between yeast strains [1]. TFs are central to decision making in cells, with roles ranging from environmental adaptation in unicellular organisms to controlling cellular differentiation and endocrine response in higher eukaryotes. TFs' unique role might have been exploited by evolution to modulate the activity of entire pathways or re-wiring of the cellular network. An example of pathway activity modulation is the shutdown of the flagellar pathway in non-motile bacterial species through the deletion of the TF activating the pathway [2]. An example of network rewiring through TF protein evolution is how a mutation in Ubx, a Hox protein, led to the loss of a subset of its targets, and is believed to have allowed the transition to a hexapod body plan [3], [4]. Attesting to the usefulness of *trans*-level variation in evolutionary adaptation is the observation that TFs underwent significantly more positive selection along the human and chimp lineages than other genes [5] and significantly more TFs had differential expression between the two species [5], [6].

The systematic mapping of molecular interactions between pairs of proteins and between proteins and DNA has unveiled a world of complexity not captured by a simple biological parts list. A useful approach to better understand protein functions and relations is to look at these networks through the lens of evolution. Evolutionary rate, typically defined as the ratio of non-synonymous to synonymous substitution rates (K_a_/K_s_), represents the level of tolerance to mutations of proteins across evolution and can reveal additional information about these networks. For example a strong trend was discovered relating protein-protein interaction (PPI) degree to evolutionary rate [7], [8], which suggests that physical interactions lead to evolutionary constraints on the protein sequence. Such genome-wide trends however ignore the underlying diversity of the many subnetworks which constitute the global network. Since genes have very distinct functions in the cell and often act together as functional modules, we might expect that the global trends not always hold for all subnetworks. Recent work by Jovelin *et al.* and by Wang *et al.* showed that the TF subnetwork evolved distinctly from the global network between closely related yeast species [9], [10]. More specifically, it was found that the number of physical interactors or transcriptional regulators correlates much more positively with evolutionary rate than is expected from the genome-wide trend. These previous studies established that subnetworks can display trends which differ significantly from those of the global network and specifically highlighted TFs as a uniquely evolving gene set.

However, no study to date has looked at how TF evolution may be influenced by other factors that are known to be important in the evolution of generic proteins, such as expression level or the evolutionary rate of network neighbors. In an effort to better understand the unique evolutionary properties of TFs, we conducted a systematic comparison of key determinants of protein evolutionary rate between TFs and generic proteins. The recent increase in the number of fully sequenced species allows us to study short term evolution, before the regulatory network has had much time to rewire. We looked at the coding sequence evolution between *S. cerevisiae* and its closest sequenced relative, *S. paradoxus*. For *S. cerevisiae*, an extensively studied model species, we have access to genome-wide protein-protein and protein-DNA interaction networks, as well as mRNA expression datasets.

Transcriptional networks display extensive evidence of modularity at the functional level. For example, it is well known that metabolic enzymes participating in a common pathway are often regulated by a common TF [11], [12]. In multicellular organisms, TFs are used to regulate tissue-specific gene expression and execute specific developmental or stimulus response programs. This functional modularity may be detectable at the evolutionary level. To test this hypothesis, we examined how TF evolutionary rate relates to the regulatory network structure, in particular how the evolutionary properties of target genes influence TF evolution. The average evolutionary properties of proteins regulated by a common TF could serve as a proxy for the amount of selective constraint acting on a transcriptional module. Since the role of a TF is defined through its transcriptional target genes and the way it regulates them, the selective constraint on a TF is expected to be proportional to the selective constraint on the transcriptional module it regulates. This network-centric function of TFs could be at the source of their distinct evolutionary trends. Since the transcriptional network is made up of a combination of activating and repressive regulatory relationships, which could have fundamentally different effects on the evolution of TFs and other genes, we also explored the effects of regulatory sign on TF-target evolutionary relationships.

## Results

### The Effect of PPI Network Degree on TF Evolution

Protein-protein interactions (PPIs), by imposing additional functions on the structure and interface residues of interacting proteins, often lead to increased selective constraints on these proteins [13]. This largely explains the empirical observation that proteins with more binding partners tend to evolve at a slower rate [7]. The slope of the correlation between network degree and protein evolutionary rate can be interpreted approximately as the average evolutionary pressure on proteins contributed by one network edge, or interaction interface. This effect has been shown to be different within the TF subnetwork than within the global network [10]. Here, we re-examined the statistical significance of the previous result using an improved method, described in detail in the Methods section, which avoids specific biases by controlling for the different average degree and evolutionary rate of TFs as compared to generic proteins. We used the ratio of the non-synonymous substitution rate (K_a_) over the synonymous substitution rate (K_s_), or K_a_/K_s_, between *S. cerevisiae* and its closest known cousin *S. paradoxus*, as a measure of protein evolutionary rate. As a normalization step, we transformed evolutionary rate and PPI degree into genome-wide ranks for all yeast protein-coding genes. We then calculated the slope for the 174 TFs (list taken from [10]) and compared it to a distribution of slopes obtained from random protein samples with the same average degree and evolutionary rate as TFs to within 1% root-mean-square deviation (RMSD). Figure S1 shows how genome-wide ranks are preserved when calculating the slope for the TF subset to allow the relative incline to be compared between TFs and generic proteins. We found that the average effect of PPIs on TF K_a_/K_s_ was significantly less pronounced than expected from the sampling procedure, with a p-value of 0.0085. Replacing the evolutionary rate with codon adaptation index (CAI), which allows us to control for the effect of expression, results in a p-value of 0.26, suggesting that expression differences are not driving the different evolutionary rate trend. Repeating the comparison using edges reported in two or more independent experiments, which we term confirmed edges (CE), returns a p-value of 0.0026, confirming that TF evolution is differentially affected by PPI degree as compared to generic proteins. The fact that the number of interaction partners does not influence TF evolutionary rate as strongly as it does for other proteins is potentially explained by the TF subnetwork's enrichment in transient interactions reusing the same binding interfaces, and depletion of stable complex formations requiring a different binding interface for each partner. Supporting this hypothesis, we used a chi-square test and the Gene Ontology (GO) [14] term “protein kinase activity” and showed that, compared to other proteins, a significantly greater fraction of TF PPIs involve kinases (2.1-fold enrichment; *p* = 1.87×10^−61^), which are known to bind transiently. Another contributing factor could be the fact that TFs, which are often bound to DNA, tend to interact with proteins which are themselves bound to DNA. The greater proximity induced by this tethering reduces the entropy of the unbound state, allowing the protein-protein interaction to be mediated by a relatively weaker binding affinity and thereby relaxing the level of selective constraint imposed by these PPI interfaces. As support for this hypothesis, we showed that a significantly greater fraction of TF PPIs are with DNA binding proteins (2.2-fold enrichment; *p* = 9.0×10^−218^), using a chi-square test and the GO term “DNA binding”.

### The Effect of Regulatory In-Degree on TF Evolution

**Figure 1:**
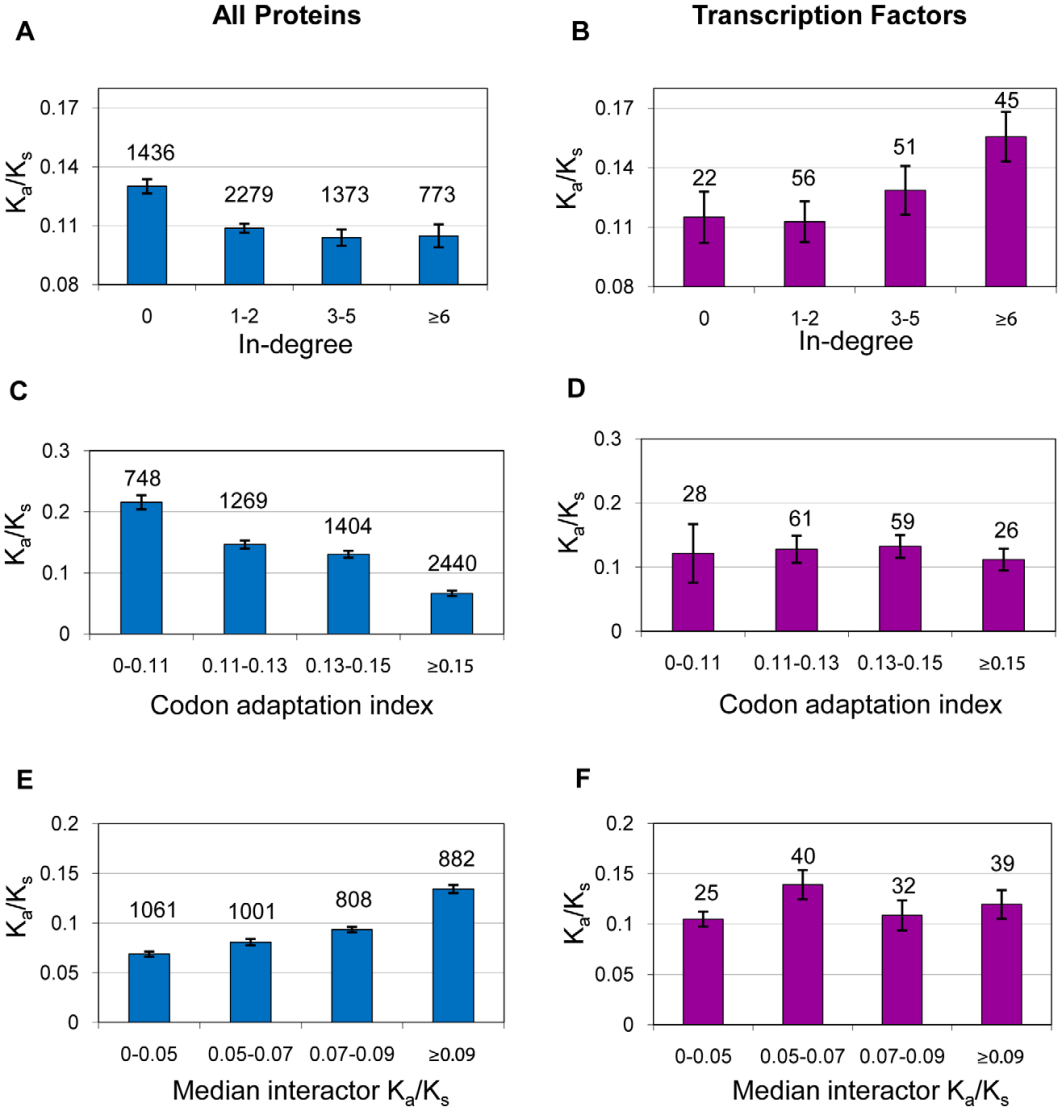
Distinct evolutionary trends of TFs. Unlike average proteins, TF K_a_/K_s_ correlates positively with regulatory in-degree and very poorly with CAI and the evolutionary rate of PPI network neighbors. K_a_/K_s_ is displayed as a function of regulatory in-degree (A–B), CAI (C–D) and median K_a_/K_s_ of interacting proteins (E–F) for all proteins (A,C,E) and TFs (B,D,F). Numbers above the bars represent the number of TFs/proteins in the bin.

Similarly to PPI degree, but with a much weaker correlation, generic proteins with more regulators (higher in-degree) tend to evolve slower. In contrast, the effect of regulatory in-degree on TFs has been shown to be opposite, with each additional regulator contributing on average towards faster evolution of the TF [10]. Using our new method and a regulatory network based on a collection of ChIP-chip studies [15], we confirmed the earlier finding that the slope relating TFs' regulatory in-degree and evolutionary rate is significantly more positive than expected by chance (*p* = 0.0093, CAI *p* = 0.042, CE *p* = 0.0041). The opposing trends relating in-degree and K_a_/K_s_ for all proteins and TFs are shown in Figure 1A and Figure 1B, respectively. To understand why high in-degree TFs tend to evolve at a faster rate, we decided to look at the genes they regulate. Although the median evolutionary rate of target genes is not significantly associated to the in-degree of regulators, we found that TFs' in-degree significantly correlates with the fraction of target genes which are missing an ortholog in the comparison species (ρ = 0.20 *p* = 0.016; CE ρ = 0.21 *p* = 0.041), *S. paradoxus*. These results suggest that the regulatory in-degree of TFs is tied to the species specificity of the transcriptional modules they regulate. High in-degree TFs may be more likely to undergo reduced negative selection than low in-degree TFs because the impairment of their regulatory functions is less likely to disrupt core processes. At the same time, high in-degree TFs may be more likely to undergo enhanced positive selection because they tend to regulate more species-specific functions.

### The Effects of Expression Level, CAI and PPI Network Neighbors on TF Evolution

Since the trends relating TF evolutionary rate to network degree are significantly distinct from the genome-wide average, we decided to probe whether TF evolutionary rate is differentially affected by other well-known correlates, such as mRNA expression level or the evolutionary rate of protein interaction partners. Using our method, we compared the slope of TFs relating K_a_/K_s_ and mRNA expression level from RNA-seq in rich media [16] to the slopes produced from random protein sets of the same size, matched for average expression level and evolutionary rate (see Methods). The results show that the trend relating TF K_a_/K_s_ to expression level is too flat to be due to chance (*p* = 0.0025), even accounting for TFs' lower average expression level. TF K_a_/K_s_ is also much less correlated than expected to CAI (*p*≤0.0001), a commonly-used surrogate for expression level. This suggests that expression level imposes weaker selective constraints on TFs than on other genes. A similar lack of correlation is also apparent between TF K_a_/K_s_ and the median K_a_/K_s_ of protein-protein interaction (PPI) partners. TF K_a_/K_s_ is too weakly correlated to that of its PPI network neighbors to be the result of chance (*p*≤0.0001). This difference is probably related to the greater fraction of TFs involved in transient interactions and thus less likely to co-evolve with their interaction partners. These results demonstrate yet again that TFs are subject to a unique set of evolutionary pressures. Figure 1 shows some of the most striking differences in TF evolutionary rate correlations. In addition to other explanations, it is possible that TF evolutionary rate shows weaker correlation to many features because of the dominant influence of other determinants on TF evolution, such as their role in the regulatory network.

### The Effect of Target Gene Evolutionary Rate on TF Evolution

**Figure 2:**
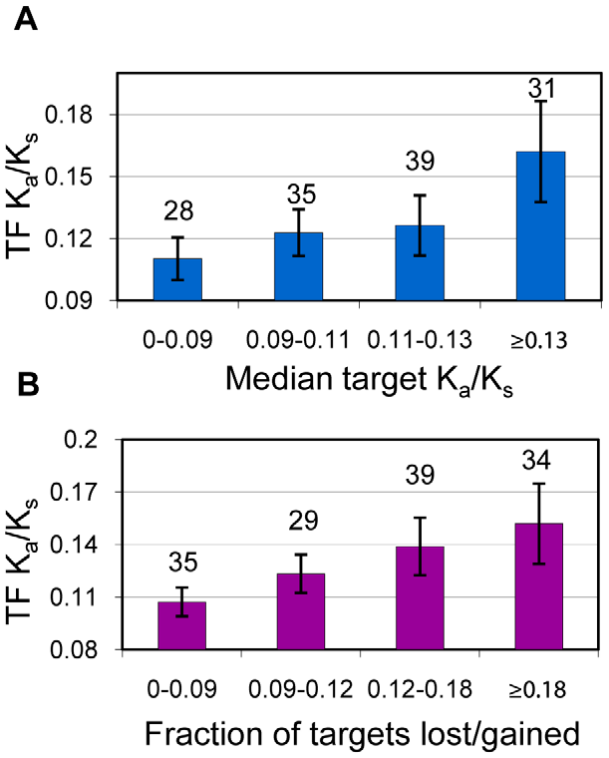
TFs and their targets co-evolve as modules. Each data point is based on a TF with 3 or more targets. (A) TF K_a_/K_s_ as a function of the median K_a_/K_s_ of target genes. (B) TF K_a_/K_s_ as a function of the fraction of target genes missing an ortholog in *S. paradoxus* (lost in *S. paradoxus* or gained in *S. cerevisiae*). Numbers above the bars represent the number of TFs in the bin.

To understand the evolutionary behavior of TFs, it is imperative that we study the evolution of their target genes. The function of TFs is inherently expressed through the regulation of their target genes and this network-centric role of TFs might be what distinguishes their evolution from that of other proteins. Using the ChIP-chip based regulatory network and Spearman's rank correlation coefficient (ρ), we asked whether median target evolutionary rate was predictive of TF evolutionary rate. As shown in Figure 2A, we discovered that the evolutionary rate of TFs significantly follows the median rate of its target genes (ρ = 0.25, *p* = 0.0033), suggesting that TFs and their target genes constitute co-evolving modules. Figure S2A shows that the correlation holds using K_a_/K_s_ values obtained from comparing *S. cerevisiae* to its next closest sequenced cousin, *S. mikatae* (ρ = 0.23, *p* = 0.0059). We also confirmed the significance of this effect using the network of confirmed edges (ρ = 0.23, *p* = 0.020) and using an alternative regulatory network based entirely on literature curation of small-scale experimental studies [15] (ρ = 0.26, *p* = 0.0018), henceforth referred to as the literature curated network. As shown in Figure S3, K_a_/K_s_ itself cannot be used to predict regulatory interactions in general, but it does provide some predictive power in the TF subnetwork (predicting TFs that regulate TFs). Furthermore, we show that targets of the same TF in the network of confirmed edges tend to have closer than expected evolutionary rates (*p* = 0.011) and mRNA expression levels (*p* = 1.13×10^−4^), using the Wilcoxon rank-sum test (see Methods for details) than targets of different TFs. Although the co-evolution of co-regulated genes is easily explained by their similar expression levels, the co-evolution of TFs and their target genes indicates that TF evolution is directly influenced by their position and role in the regulatory network.

### The Effect of Target Gene Loss or Gain on TF Evolution

Evolutionary rate is not the only evolutionary measure of protein importance. We also looked at the fraction of target genes missing an ortholog in the closest yeast species, *S. paradoxus*, indicating the gene was either lost in *S. paradoxus* or gained in *S. cerevisiae*. We discovered that the fraction of target genes missing in *S. paradoxus* is correlated to TF evolutionary rate (ρ = 0.22, *p* = 0.0091; Figure 2B). The correlation was confirmed using *S. mikatae* as the comparison species (ρ = 0.23, *p* = 0.0081), as displayed in Figure S2B. The result also holds using the network of confirmed edges (ρ = 0.24, *p* = 0.021) and the alternative literature curated network (ρ = 0.24, *p* = 0.0042). These results suggest that the evolutionary rate of TFs is tied to the species specificity of the transcriptional modules they regulate. TFs regulating species-specific modules tend to evolve faster, as a result of either relaxed negative selection or enhanced positive selection.

### The Relative Contribution of TF Evolutionary Rate Correlates

**Figure 3:**
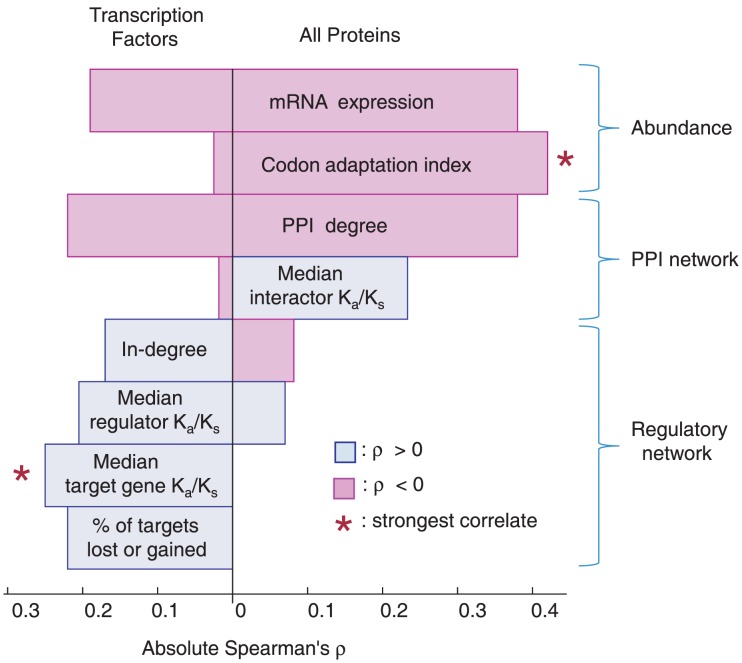
Comparison of different genomic and network features influencing TF and protein evolutionary rate. For each determinant, absolute Spearman's rank correlation coefficient (ρ) for TFs is displayed on the left and for all proteins, on the right, with the color of the box representing the direction of the trend. The * indicates the most dominant correlation for each protein set. While CAI is the dominant correlate with K_a_/K_s_ for generic proteins, target gene K_a_/K_s_ is the strongest correlate for TF K_a_/K_s_.

In addition to separately assessing the genomic and network correlates of TF evolutionary rate, it is important to compare their relative contributions to identify the most dominant determinants of TF evolutionary rate, and whether they differ from those of generic proteins. Figure 3 shows the Spearman's rank correlation coefficients (ρ) relating different genomic and network properties to K_a_/K_s_ for TFs and for all proteins. This figure clearly shows how features like expression, CAI, which is tightly coupled to expression [17], and PPI degree dominate the evolutionary rate determinant landscape of average proteins. In contrast, median target K_a_/K_s_ dominates the TF landscape, with other regulatory network properties playing an important role, such as in-degree, median regulator K_a_/K_s_ and the fraction of target genes missing in *S. paradoxus*. This shows that the regulatory network structure is the most important factor determining TF evolutionary rate, suggesting that the function and evolution of TFs is primarily defined at the network level. The dominance of this so far overlooked relationship between TF and target evolution could also potentially explain the eccentricity of other TF evolutionary trends. The observation that TFs have significantly different evolutionary rate determinants was confirmed individually for each variable earlier in the Results section, using sampling of random proteins and rigorous statistical tests as described in the Methods section.

In contrast to random samples, other functionally defined subsets of proteins may also possess a different landscape of evolutionary rate determinants. As case examples, Figure S4 shows the same evolutionary rate determinant correlation coefficients for the 240 proteins in the GO term “signal transduction” and for 540 metabolic enzymes taken from the YeastCyc database [18]. We see that these functionally defined categories have similar overall evolutionary rate determinant profiles to that of generic proteins in Figure 3, with abundance and PPI degree dominating the landscape, suggesting that TFs are unique in this regard among functionally defined protein subsets. The only notable exception is the lack of correlation between signal transduction protein K_a_/K_s_ and its median interactor K_a_/K_s_, which is consistent with our theory that this effect in TFs may be related to the transience of many interactions in the subnetwork.

### Controlling for Potential Relationships between Other TF and Target Properties

Since target K_a_/K_s_ is apparently the strongest determinant of TF evolutionary rate, it is important to look for potential relationships between key TF and target properties, such as mRNA expression level and PPI degree, to rule out potential confounding effects. Table S1 shows the Spearman's rank correlation coefficients relating different TF and target properties. We have repeated each one of these correlations using the network of confirmed edges and using the literature curated network [15], the results of which are shown in Tables S2 and S3, respectively. This analysis reveals that median target K_a_/K_s_ remains the strongest predictor of TF K_a_/K_s_ over other important target properties.

Since TFs are often regulated post-translationally, target gene expression has been used in studies to estimate the level of TF activity [19], [20]. Although consistently negative, the correlation between target expression and TF K_a_/K_s_ was only found to be significant using the literature curated network. This result suggests that TF activity as estimated from target gene expression cannot be the only driving force behind the modularity of TF-target evolution. Further studies are needed to investigate the role of TF activity in determining TF evolutionary rate.

### TF Evolution and Target Gene Function

Having found that TF evolutionary rate is related to the evolutionary rate and species-specificity of target genes, we may expect a similar relation between TF evolutionary rate and target gene function. We looked for enrichments of large GO terms (involving 50 or more target genes) in the targets of the 25% fastest evolving TFs with targets, as compared to targets of other TFs using Fisher's exact test. Table 1 shows the enrichments with a p-value below 0.05. Most GO terms that were significantly enriched in targets of fast evolving TFs are indicative of niche-specific functions, such as transporter activity, oxidation-reduction processes, and localization to the extracellular region, plasma membrane or cell periphery, as well as categories likely to show niche-specific expression, like carbohydrate metabolism. Most interestingly, we found that fast evolving TFs were also more likely to regulate other TFs, suggesting that the hierarchical structure of the regulatory network may be exploited by adaptive evolution. Table S4 shows the enrichments found in targets of the other, slower evolving, TFs for comparison. It features terms representing more central and environment-independent functions, such as ribosomal or RNA processing functions and intracellular, organellar or nuclear localization. These results suggest that TF evolution potentially serves as a mechanism for species-specific environmental adaptation through its effect on the expression of multi-gene modules.

**Table 1:** Go terms significantly enriched in targets of 25% fastest evolving TFs as compared to targets of other TFs.

Functional Term	GO ID	# of Genes	Fold enrichment	p-value
fungal-type cell wall	GO:0009277	75	1.56	0.00014
cell wall	GO:0005618	77	1.54	0.00022
external encapsulating structure	GO:0030312	77	1.54	0.00022
cell periphery	GO:0071944	313	1.19	0.0036
extracellular region	GO:0005576	63	1.43	0.0061
plasma membrane	GO:0005886	214	1.22	0.0078
oxidoreductase activity	GO:0016491	172	1.24	0.012
carbohydrate metabolic process	GO:0005975	156	1.25	0.017
transporter activity	GO:0005215	213	1.20	0.018
transmembrane transporter activity	GO:0022857	178	1.21	0.020
substrate-specific transmembrane transporter activity	GO:0022891	162	1.22	0.021
transcription factors, as taken from [10]	NA	102	1.28	0.024
substrate-specific transporter activity	GO:0022892	190	1.19	0.026
ion transmembrane transporter activity	GO:0015075	89	1.29	0.031
sequence-specific DNA binding	GO:0043565	123	1.24	0.033
ion transmembrane transport	GO:0034220	93	1.27	0.035
alcohol metabolic process	GO:0006066	102	1.28	0.036
carbohydrate biosynthetic process	GO:0016051	51	1.42	0.036
transmembrane transport	GO:0055085	204	1.17	0.046

### TF Evolution and the Evolution of Target Gene Expression

**Figure 4:**
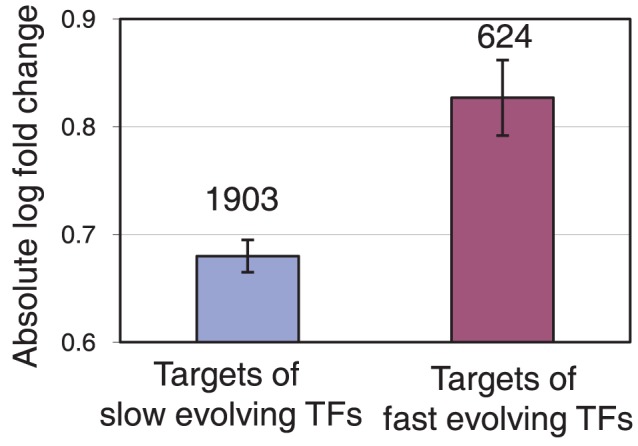
Targets of fast evolving TFs have larger expression changes through evolution. The targets of the 25% fastest evolving TFs, on the right, have on average larger absolute fold changes in expression between *S. cerevisiae* and *S. paradoxus* than targets of other TFs, on the left, as determined by RNA-seq. Numbers above the bars represent the number of TFs in the bin.

The role of *trans*-regulatory gene evolution on gene expression is inherently more difficult to study than *cis*-regulatory evolution since the former requires knowledge of the regulatory network structure. To confirm that the evolutionary rate of TFs is related to measurable *trans*-regulatory changes in the gene expression of target genes, we used previously published RNA-seq data from both *S. cerevisiae* and *S. paradoxus* [21]. Using the network of confirmed ChIP-chip edges, we found that targets of the top 25% fastest evolving TFs had, on average, larger expression differences between the two species than targets of other TFs, as shown in Figure 4 (t-test *p* = 0.00013, see Methods for details). This result confirms that TF evolutionary rate can serve to predict real *trans*-regulatory expression changes of gene modules, which could in turn lead to important phenotypic effects.

### The Effect of Regulatory Sign on TF-Target Co-evolution

**Figure 5:**
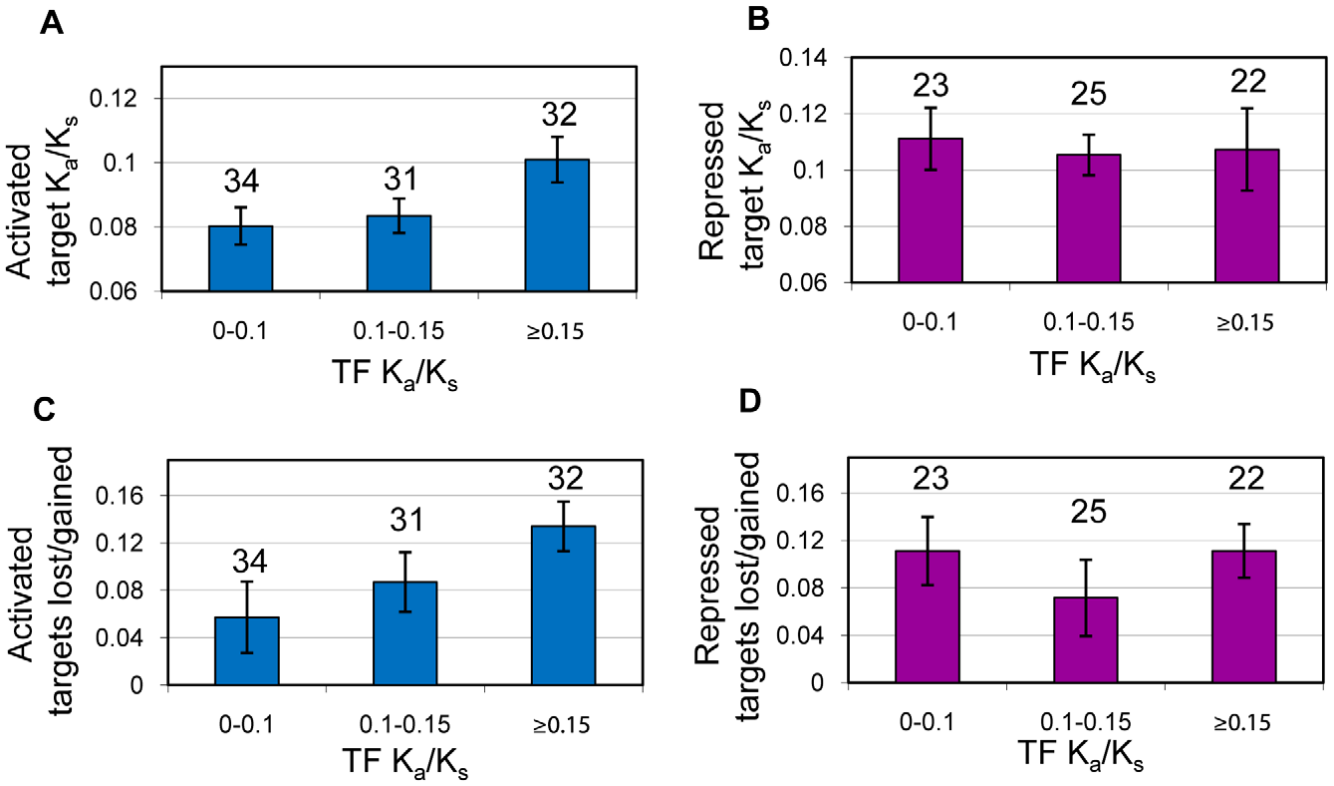
TFs co-evolve with activated targets, but not with repressed targets. Edge signs are inferred from TF knock-out expression data. Each data point is based on a TF with 5 or more targets regulated in the same direction. (A) Median K_a_/K_s_ of activated target genes as a function of TF K_a_/K_s_. (B) Median K_a_/K_s_ of repressed target genes as a function of TF K_a_/K_s_. (C) Fraction of activated targets missing an ortholog in *S. paradoxus* as a function of TF K_a_/K_s_. (D) Fraction of repressed targets missing an ortholog in *S. paradoxus* as a function of TF K_a_/K_s_. Numbers above the bars represent the number of TFs in the bin.

Regulatory networks are composed of two inherently distinct edge types, activating (or positive) edges and repressive (or negative) edges, which could potentially play divergent roles on the evolutionary modularity of the network. We used previously published TF knock-out microarray data [22] to infer the sign of ChIP-chip based regulatory network edges. Using the microarray fold-changes (see Methods for details), we were able to infer the mode of regulation for 4,010 of the ChIP-chip regulatory edges, 2,628 activating and 1,382 repressive. By overlaying these two datasets, we decomposed the network into positive and negative regulatory subnetworks and studied how the mode of regulation affects TF-target evolutionary relationships. For TFs with 5 or more targets of the same regulatory sign, we found that median K_a_/K_s_ of activated targets significantly follows TF K_a_/K_s_ (ρ = 0.26, *p* = 0.0036), while median K_a_/K_s_ of repressed targets shows no significant correlation (ρ = 0.068, *p* = 0.46). We also found that TF K_a_/K_s_ predicts the fraction of activated targets which are missing in the comparison species *S. paradoxus* (ρ = 0.29, *p* = 0.0038) but not for repressed targets (ρ = −0.079, *p* = 0.52). Table 2 shows the correlation coefficients and associated p-values for activating and repressive networks, where transcriptional edges are inferred either from ChIP-chip or from literature curation of small-scale experimental studies [15]. As shown in Table 2, both the significance of the activating edge relations and the lack of a significant trend for repressive edge relations were confirmed using the literature curated network. Figure 5 shows how activated and repressed target evolutionary properties have a different effect on TF K_a_/K_s_. These results demonstrate that TFs evolve in synchrony with the targets they activate but not the targets they repress.

**Table 2:** Correlations between TF K_a_/K_s_ and evolutionary properties of activated or repressed target genes.

		ChIP-chip Network		Literature-curated Network	
Regulatory Sign	Property	Median target K_a_/K_s_	Fraction of targets lost or gained	Median target K_a_/K_s_	Fraction of targets lost or gained
Activated	rho (ρ)	0.26	0.29	0.31	0.39
	p-value	0.0036	0.0038	0.00039	0.00029
Repressed	rho (ρ)	0.068	−0.079	0.10	0.065
	p-value	0.46	0.52	0.30	0.61

## Discussion

Protein sequence evolutionary rate provides a unique viewpoint into both the importance and the functional relationships between genes and proteins. In this study, we have demonstrated how the function of TFs in the regulatory network is more important in understanding TF evolution than any other property measured. Demonstrating how TF sequence evolution plays an important role in the evolution of gene expression, we have shown that targets of fast evolving TFs are more likely to see their expression change through evolution. We found that TF evolutionary rate is determined by very different rules than that of generic proteins, possessing a unique correlation to expression level, CAI, median evolutionary rate of PPI network neighbors and, as previously reported, to PPI degree and regulatory in-degree. This evidence demonstrates how TFs are subject to their own set of evolutionary pressures.

We have also demonstrated that TF evolutionary rate is strongly related to the evolutionary properties of their target genes, such as evolutionary rate and species-specificity. Remarkably, this network-level influence on TF evolutionary rate trumps even that of gene expression. The fact that TFs and their targets tend to evolve as modules is consistent with similar findings in other types of biological networks. It has previously been reported that neighbors in many types of biological networks tend to evolve at similar rates [23], including PPI networks [7], co-expression networks [24], genetic interaction networks [25] and metabolic networks [26]. What we have demonstrated here is that neighbor genes also tend to evolve at similar rates in the transcriptional network (TFs and their targets) and co-regulation network (genes regulated by a common TF). It is important to note, since TFs have low expression and are often regulated post-translationally, that the regulatory network is the one network among these (including the co-regulation network) for which the co-evolution of protein sequences is the least likely to be explained by similar expression levels.

The lack of correlation with CAI and expression level is especially surprising since it has been thoroughly established that protein abundance is by far the strongest predictor of protein evolutionary rate [7], [27], [28]. This is believed to relate to the increased pressure for proper folding and translational accuracy in highly expressed proteins [29]. Since TFs' distinct trend is not explained by their lower average expression level, the difference is likely related to TFs' cellular role. Their expression levels may be subject to more variation across species, as shown across the human-chimp lineage [6]. TFs could also be subject to other dominant evolutionary constraints, such as their network-level role.

Looking for unique features of TFs which could explain their distinct evolutionary trends, we found evidence suggesting TFs may play a special role in adaptive evolution. We have shown that targets of fast evolving TFs are more likely to show differential expression between the two yeast species and that targets of these same TFs are also more likely to be involved in environment-specific functions. TFs are themselves more likely to be regulated by fast evolving TFs, suggesting the possibility that adaptive evolution has taken advantage of the hierarchical structure of the regulatory network to achieve desirable phenotypic changes more efficiently.

Another strikingly unique feature of TF evolution is the positive trend relating TF evolutionary rate to regulatory in-degree, while other proteins show a negative trend. Here, we found that this positive trend appears to be a module-level trend, with TF in-degree affecting not only the TFs evolutionary rate but also the species-specificity of genes regulated by those TFs. The fact that TFs were more likely to be regulated by fast evolving TFs than other genes could also help explain this trend, especially considering that TF evolutionary rate is much more sensitive to the evolutionary rates of their regulators than is the case for other proteins, as shown in Figure 3. To gain further insights into the relation between TF in-degree and target function, we calculated the enrichment of GO term memberships comparing targets of high in-degree TFs (≥10 regulators) to targets of low in-degree TFs (≤2 regulators) using Fisher's exact test, considering GO terms with 50 or more targets. As shown in Table S5, the GO terms that were significantly enriched for targets of high in-degree TFs are very similar to those of fast evolving TFs, centering around peripheral or niche-specific functions, such as plasma transmembrane transport, while GO terms enriched in targets of low in-degree TFs, shown in Table S6, represent more central and environment-independent functions, such as translation and primary metabolism. These results suggest that TFs regulating niche-specific genes tend to have higher in-degree in part to allow for the integration of environmental signals.

When we decomposed the regulatory network into positive and negative regulatory subnetworks, we found that only positive regulatory relationships predict co-evolution of TFs and their targets. A study by Hershberg *et al.* supports that there are distinct evolutionary pressures on activator and repressor TFs in relation to their role in the transcriptional network. They discovered by comparing different strains of bacteria that activators are more likely than repressors to be lost before all their targets are lost [2]. They suggested that the loss of activator TFs was an “efficient means of shutting down unused pathways”. This draws a picture where activator TFs can be used by evolution as on/off switches affecting the activity of multi-gene modules, thereby avoiding the need to silence each gene through individual mutations. The loss of repressors however tends to be avoided regardless of the usefulness of the genes they regulate. Losing a repressor would likely lead to the untimely expression of genes, which will incur an energetic cost and potentially disrupt homeostasis. Similarly to the loss of a TF, mutations in the protein sequence of a TF are likely to impair the function of that TF. In the case of an activator, this would lead to reduced expression of regulated genes. We therefore expect the more conserved genes modules to be regulated by more conserved TFs, and vice-versa. In the case of a repressor, mutations in its protein sequence would likely lead to the over-expression of target genes, which due to resource expenditure and/or dosage sensitivity can be damaging to the cell independently of the evolutionary importance of target genes. This would explain why the evolutionary rate of repressors is largely independent of the evolutionary properties of target genes. Our results are consistent with Hershberg *et al.*'s earlier findings, but suggest that the loss of activator TFs is an extreme example within the wider spectrum of activator TF protein evolution, which can likely be involved in more subtle and varied modulations of pathway activities than a simple on/off switch. This new perspective on the evolution of *trans*-regulatory gene expression control confirms that positive and negative regulatory subnetworks are subject to very different evolutionary pressures at the regulatory network-level.

This work details the uniqueness of TF evolutionary rate determinants and is the first to establish the modularity of TF-target protein evolution. This new awareness sheds much needed light on the eukaryotic evolution of *trans*-level control of gene expression through TF protein evolution and may help us better understand how subtle differences at the protein level can lead to pathway level variation between species. We also demonstrated that there are fundamental evolutionary differences between positive and negative regulatory subnetworks. Identifying consistent themes in the ways regulatory networks achieve favorable adaptations can reveal design principles underlying the system's dynamics and evolutionary adaptability. On a wider note, this work has established that for a subset of proteins, systems-level properties can leave evolutionary traces of comparable effect size to physical features such as expression level and PPI degree.

## Methods

### Data Collection

We used the yeast ChIP-chip data available from the YEASTRACT database (http://yeastract.com) [15] compiled from multiple studies [30]–[35]. The literature-curated transcriptional network dataset, which is based on small-scale experimental studies, was also retrieved from the YEASTRACT database [15]. We downloaded physical interaction data from the *Saccharomyces* Genome Database (SGD) [36], which compiled PPIs from different high-throughput and small-scale studies. Orthology between *S. cerevisiae* and *S. paradoxus* were taken from the Orthogroups database (http://www.broadinstitute.org/regev/orthogroups/) [37]. K_a_/K_s_ values were calculated according to the Yang-Neilsen method [38] using PAML [39]. Genes missing an ortholog were assigned a K_a_/K_s_ value higher than the fastest evolving genes with an ortholog. Codon adaptation index (CAI) values were taken from Wang *et al.* [10]. TF knock-out microarray expression data [22] (accession #: GSE4654) and RNA-seq expression data [16], [21] (accessions: GSE13750 and GSE32679) were retrieved from the Gene Expression Omnibus database (http://www.ncbi.nlm.nih.gov/geo/).

### Calculating and Comparing Slopes

To allow for the comparison of slopes between TFs and all proteins, without succumbing to the pitfalls associated with the use of highly non-normal distributions, we developed a new normalization procedure. We simply assign ranks to all proteins in the original, genome-wide, distribution. Then we use these ranks to calculate slopes on the different protein sets, rather than re-ranking within the subsets as would Spearman's rank correlation. The problem with re-ranking within the subsets is that the slopes will be normalized to equal the correlation coefficient, which represents the goodness of fit rather than the relative slope. This modified procedure allows us to compare the degree of the slope between the TF subset to the global protein set.

### Assigning P-values to Subnetwork Slopes

We calculated a p-value for the unexpectedness of the TF slope as compared to the slope for generic proteins, using a sampling procedure similar to the approach used in [10]. We produced a distribution of 10,000 slopes by performing regressions on randomly selected equally sized samples of proteins whose average K_a_/K_s_ and degree (or the relevant pair of variables) in rank space are within 1% root-mean-square deviation (RMSD) of the TF subset. P-value is calculated as the fraction of slopes generated from random samples whose incline is more extreme than or equal to that of the slope associated with the TF subset. It is essential to control for average rate and degree because having a different distribution in either dimension can systematically bias the slope. As compared to the method applied in [10], our new method differs in that we used directly comparable slopes obtained from the genome-wide rank space instead of the correlation coefficient, and in that we controlled for the different average evolutionary rate (and other relevant variables) of TFs as compared to generic proteins. This improved method allows us to draw conclusions in more confidence, having excluded additional potential confounding factors.

### Histograms and Error Bars

For each histogram, we plotted the median value for each bin, which is more robust to outliers than the average, and used bootstrapping with a 100 re-samplings to estimate the standard error of the median. Using the median rather than the mean also produces results which are insensitive to the choice of K_a_/K_s_ assigned to genes which lack an ortholog in the reference species *S. paradoxus*.

### Controls

As a control for the K_a_/K_s_ to PPI degree and in-degree trend comparisons, we repeated the calculations, replacing the evolutionary rate of each protein with its codon adaptation index (CAI), which is considered a good proxy for the average expression level [17]. This way, we can confirm or discard the hypothesis that a surprising slope relating evolutionary rate and degree is explained by a different trend relating the strong correlate, expression, to degree. This approach was used previously in [10]. To ensure that false positive interactions are not a problem, we also repeated these correlations using only network edges which are supported by two or more independent ChIP-chip experiments, which we termed confirmed edges (CE).

### Measuring the Relationship between TF and Target Properties

For every TF with 3 or more targets, based on ChIP-chip edges, we measured the median K_a_/K_s_, the fraction of targets missing a *S. paradoxus* ortholog as well as the fraction of highly conserved, highly interactive and highly expressed targets (top 20%) and used Spearman's rank correlation to establish the significance of the correlations. We repeated the analysis using literature derived edges and using only confirmed ChIP-chip edges (CE). We used TFs with 2 or more targets for the analysis with confirmed edges, since the resulting network is sparser and edges more reliable. In the case of the fraction of targets missing an ortholog, we still required at least 3 targets because this feature affects a small fraction of genes (∼10%). We considered robust the correlations which were found to be significant (*p*<0.05) in all three networks. For activating and repressive networks, we used TFs with 5 or more targets regulated in the same direction to ensure the correlations are robust to the potential uncertainties in the sign of regulatory edges.

### Calculating the Spread of K_a_/K_s_ and Expression of Co-regulated Genes

For each TF with 3 or more targets possessing an ortholog in *S. paradoxus*, we calculated the median K_a_/K_s_ and mRNA log read count difference between all pairs of targets and compared the result to the expected difference using the Wilcoxon rank-sum test. The expected median difference was estimated from the average of 100 equally-sized randomized sets of “target” genes, where each gene was chosen with a probability proportional to its in-degree.

### Comparing the Expression-Level Differences of Genes between Two Yeast Species

We used previously published RNA-seq data from both *S. cerevisiae* and *S. paradoxus* [21] to measure the extent of gene expression change through evolution. We first normalized expression levels by dividing the number of reads mapping to each gene by the number of millions of reads in the sample (reads-per-million), fixing the lowest possible gene expression at 1 read-per-million. This procedure controls for differences in sequencing depth, allowing the levels for each gene to be comparable across the two species. We then measured the log_2_ fold expression change between the two species for each gene, using the orthology assignments provided by the expression study. Using logged values makes the fold change distribution closer to a normal distribution. We then used an unpaired t-test to determine the significance of the difference between absolute log_2_ fold changes of targets of fast evolving TFs (top 25%) and targets of slow evolving TFs (bottom 75%).

### Assigning Signs to Regulatory Edges

To assign a positive or negative sign to regulatory edges, we used previously published TF knock-out microarray data [22] which includes 135 TFs with ChIP-chip data. For ChIP-chip derived edges which corresponded to an X score [22], a confidence-weighted log ratio, of absolute value greater than 1, we inferred the sign of the edge based on the target gene expression change. The same approach was used for literature-based edges.

## Supporting Information

Figure S1 **Scatter plots for distinct evolutionary trends of TFs compared to generic proteins.** Shown are rank-rank plots and trend lines for all proteins (in blue) and TFs (in purple), where K_a_/K_s_ is displayed as a function of regulatory in-degree (A), PPI degree (B), median K_a_/K_s_ of interacting proteins (C), and CAI (D).
(PDF)

Figure S2 **TF-target co-evolution between** ***S. cerevisiae*** **and** ***S. mikatae*** **.** (A) Median K_a_/K_s_ of target genes as a function of TF K_a_/K_s_. (B) Fraction of targets missing an ortholog in *S. mikatae* (lost in *S. mikatae* or gained in *S. cerevisiae*) as a function of TF K_a_/K_s_. Numbers above the bars represent the number of TFs in the bin.
(PDF)

Figure S3 **K_a_/K_s_ as predictor for transcriptional regulation.** Shown are the Receiver Operating Characteristic (ROC) curves over the entire ChIP-chip network (A) and the TF subnetwork (B) of regulatory interaction prediction based on linear regression between TF K_a_/K_s_ and median target K_a_/K_s_. In each case, TFs were randomly split into a training set, on which regression was performed, and a test set, on which true positive and false positive rates were assessed. The figure shows that K_a_/K_s_ does not predict regulatory edges in the global network, but it does provide some predictive power when limited to the TF subnetwork (TFs regulating TFs).
(PDF)

Figure S4 **Comparison of different genomic and network features influencing evolutionary rate of metabolic enzymes and signal transduction proteins.** For each determinant, absolute Spearman's rank correlation coefficient (ρ) is displayed, with the color of the box representing the direction of the trend. (A) Evolutionary rate determinants of 540 metabolic enzymes taken from YeastCyc. (B) Evolutionary rate determinants of the 240 proteins in the GO term “signal transduction”. This figure shows that functionally defined protein sets other than TFs have evolutionary rate determinant profiles similar to that of generic proteins.
(PDF)

Table S1 **Spearman correlation coefficients relating TF and target properties in the ChIP-chip network.**
(DOC)

Table S2 **Spearman correlation coefficients relating TF and target properties in the network of confirmed edges.**
(DOC)

Table S3 **Spearman correlation coefficients relating TF and target properties in the network of literature curated edges.**
(DOC)

Table S4 **Go terms significantly enriched in targets of 75% slowest evolving TFs as compared to the targets of 25% fastest evolving.**
(DOC)

Table S5 **GO terms significantly enriched in target genes of TFs with 10 or more regulators as compared to targets of TFs with 2 or less regulators.**
(DOC)

Table S6 **GO terms significantly enriched in target genes of TFs with 2 or less regulators as compared to targets of TFs with 10 or more regulators.**
(DOC)
