# Supplementary figures and images for: Regulatory Network Structure as a Dominant Determinant of Transcription Factor Evolutionary Rate
Source: PLoS Comput Biol. 2012 Oct 18;8(10):e1002734. doi: 10.1371/journal.pcbi.1002734 (PMC3475661; doi:10.1371/journal.pcbi.1002734)

A

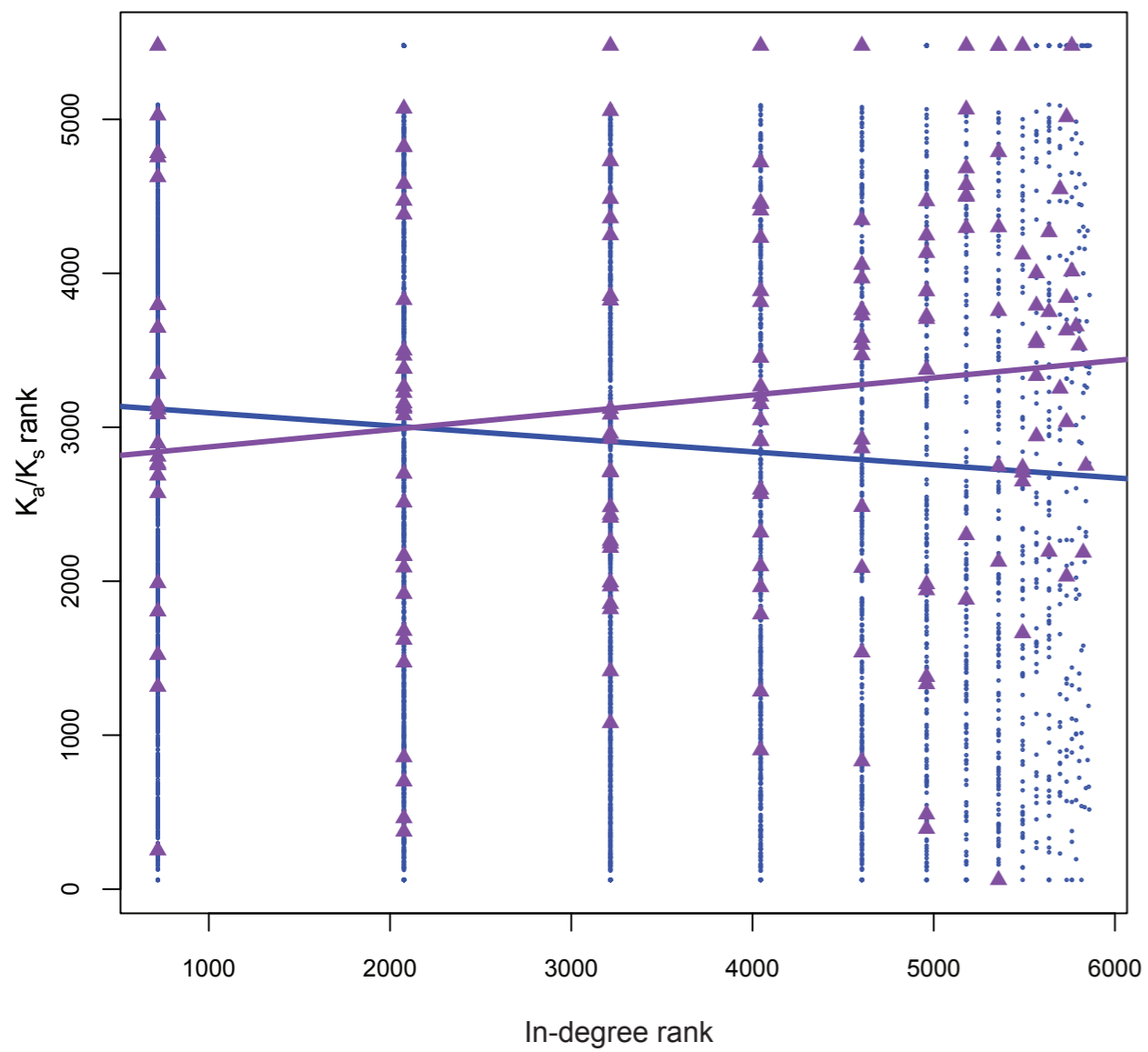

B

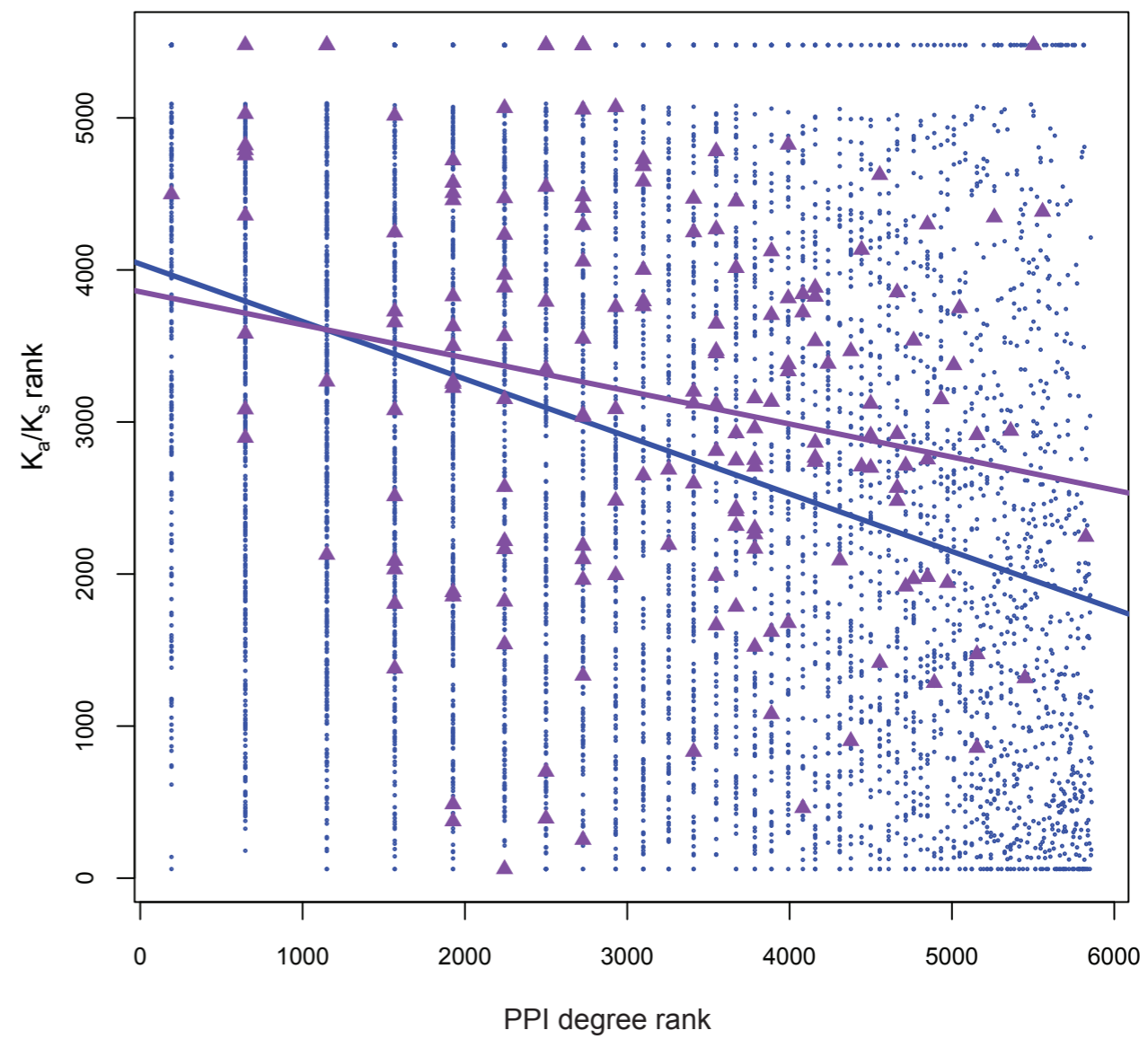

C

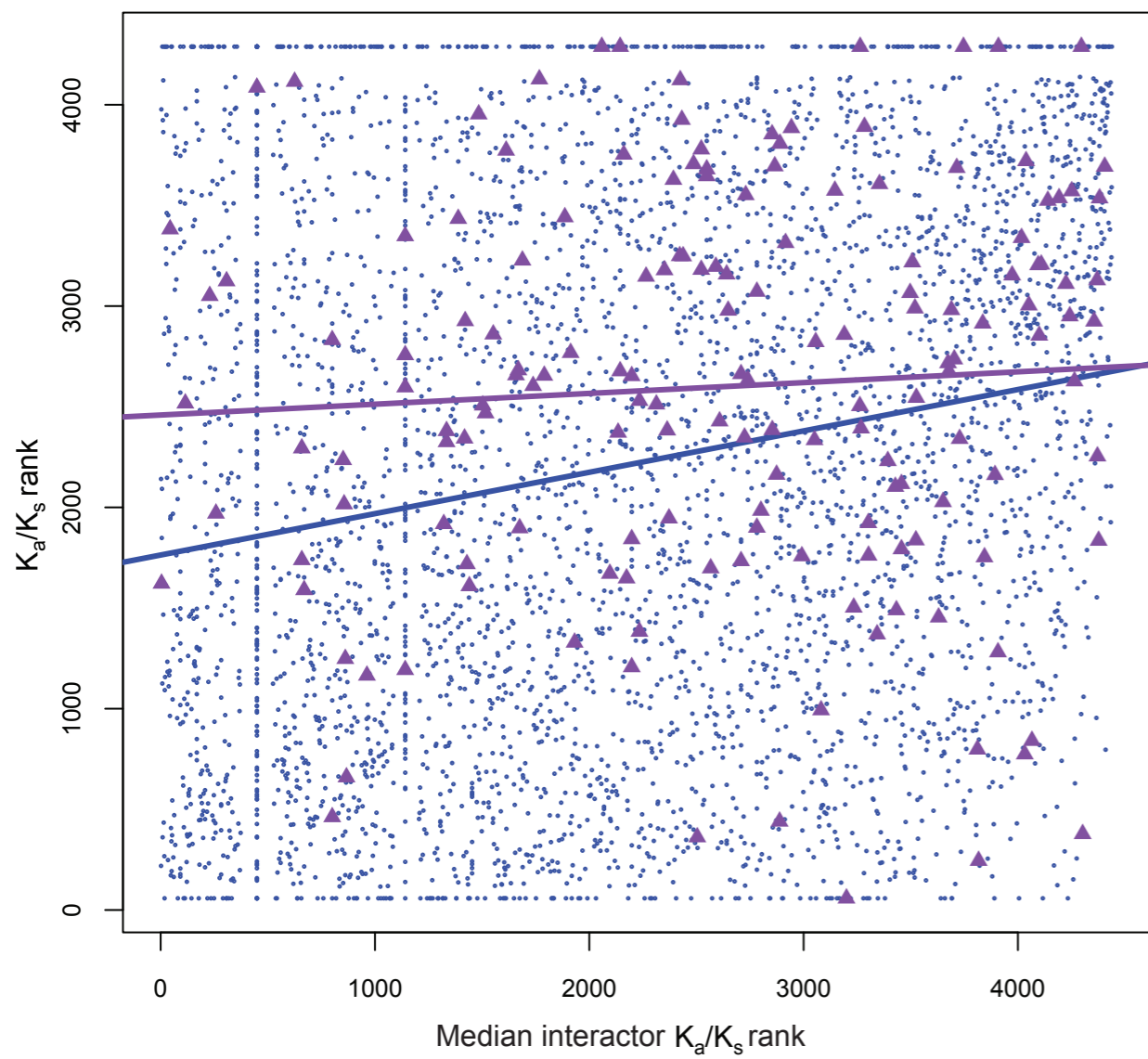

D

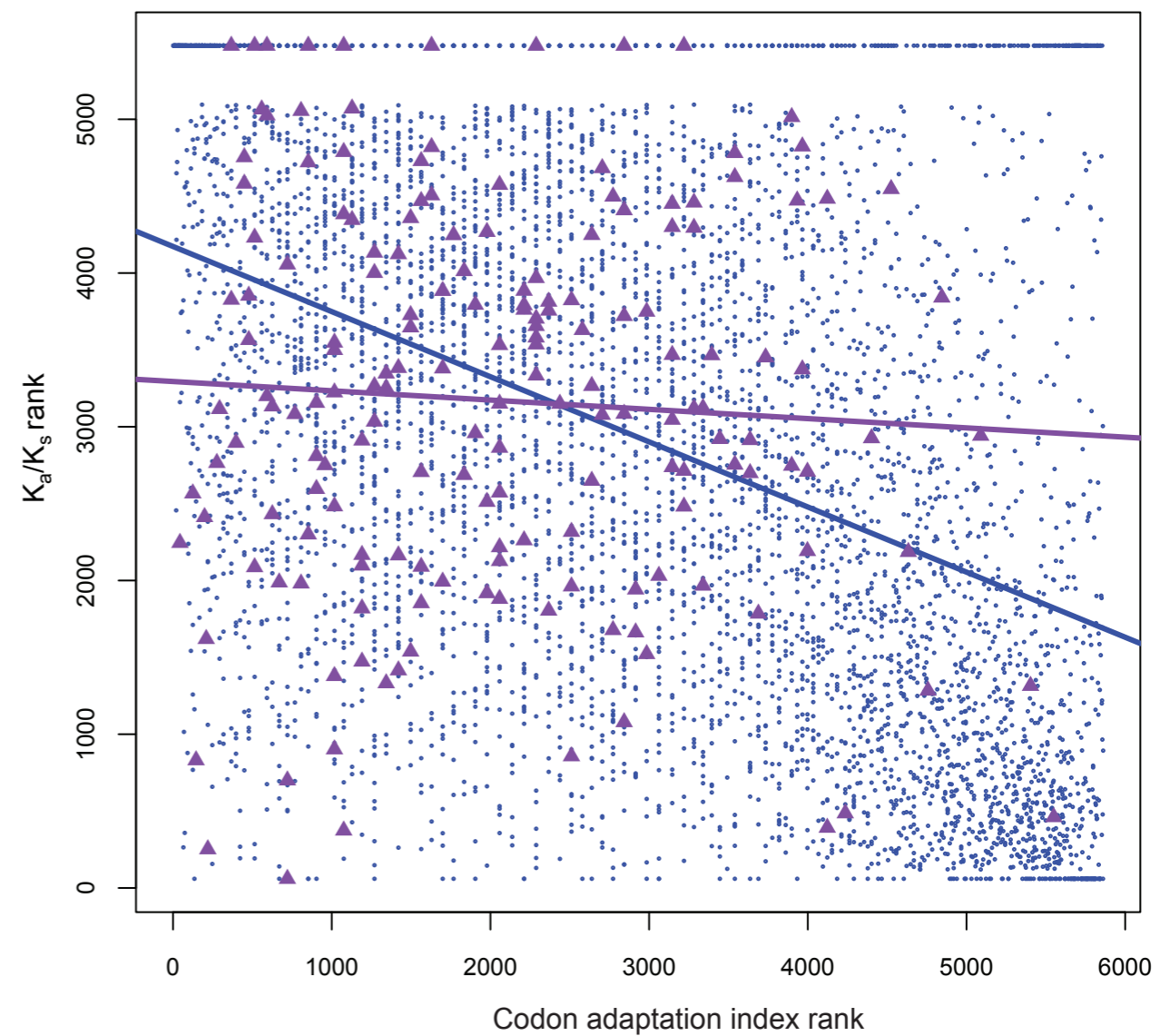

● : All proteins    ▲ : Transcription factors

Supplement: Figure S1 — Scatter plots for distinct evolutionary trends of TFs compared to generic proteins. Shown are rank-rank plots and trend lines for all proteins (in blue) and TFs (in purple), where Ka/Ks is displayed as a function of regulatory in-degree (A), PPI degree (B), median Ka/Ks of interacting proteins (C), and CAI (D). (PDF) [file pcbi.1002734.s001.pdf]

**A**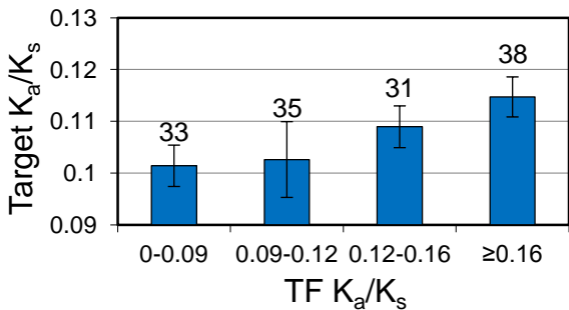**B**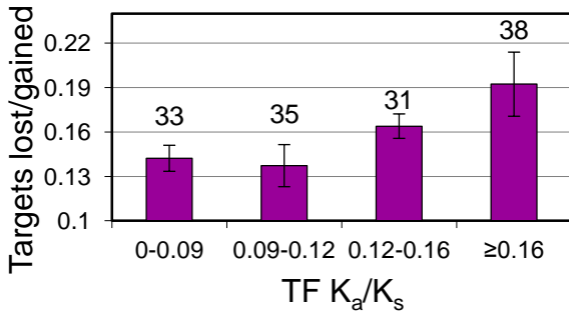

Supplement: Figure S2 — TF-target co-evolution between S. cerevisiae and S. mikatae . (A) Median Ka/Ks of target genes as a function of TF Ka/Ks. (B) Fraction of targets missing an ortholog in S. mikatae (lost in S. mikatae or gained in S. cerevisiae) as a function of TF Ka/Ks. Numbers above the bars represent the number of TFs in the bin. (PDF) [file pcbi.1002734.s002.pdf]

**A**

Genome-wide Network

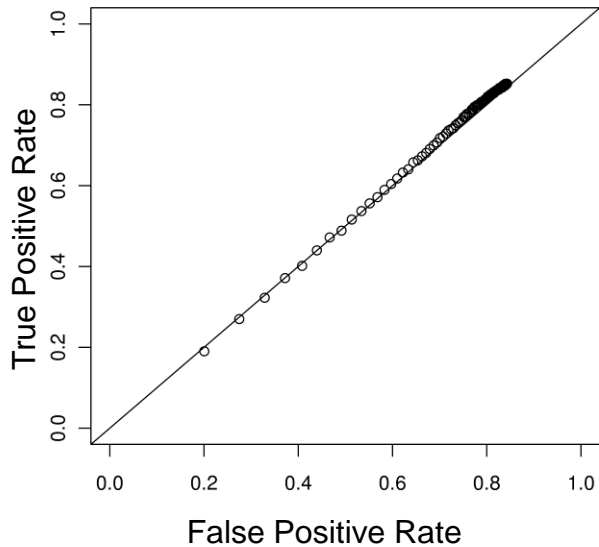**B**

Transcription Factor Subnetwork

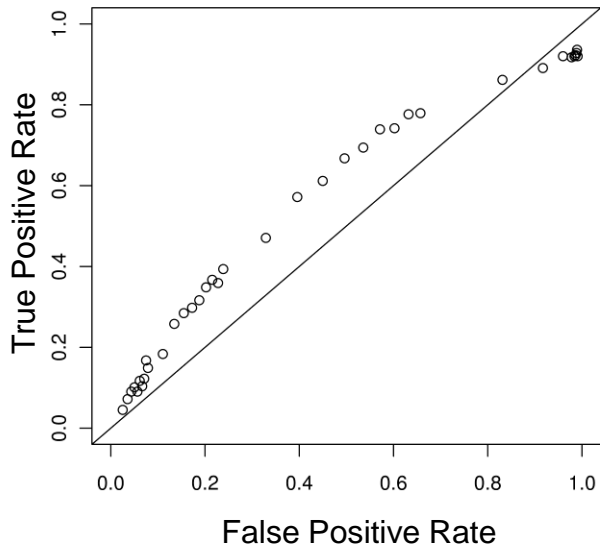

Supplement: Figure S3 — Ka/Ks as predictor for transcriptional regulation. Shown are the Receiver Operating Characteristic (ROC) curves over the entire ChIP-chip network (A) and the TF subnetwork (B) of regulatory interaction prediction based on linear regression between TF Ka/Ks and median target Ka/Ks. In each case, TFs were randomly split into a training set, on which regression was performed, and a test set, on which true positive and false positive rates were assessed. The figure shows that Ka/Ks does not predict regulatory edges in the global network, but it does provide some predictive power when limited to the TF subnetwork (TFs regulating TFs). (PDF) [file pcbi.1002734.s003.pdf]

**A**

## Metabolic enzymes

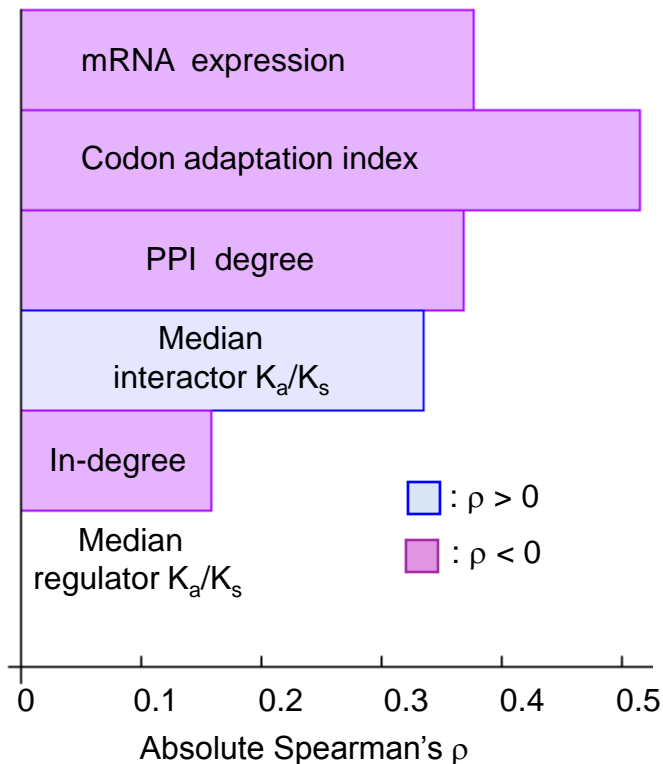**B**

## Signal Transduction

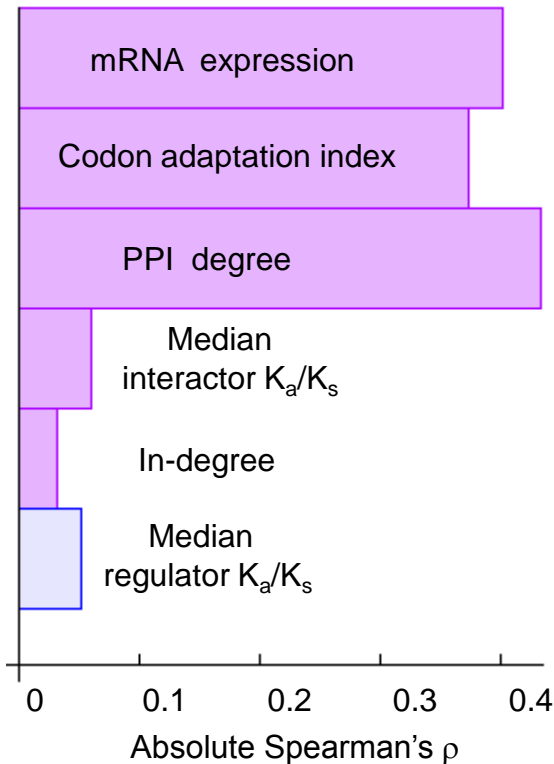

Supplement: Figure S4 — Comparison of different genomic and network features influencing evolutionary rate of metabolic enzymes and signal transduction proteins. For each determinant, absolute Spearman's rank correlation coefficient (ρ) is displayed, with the color of the box representing the direction of the trend. (A) Evolutionary rate determinants of 540 metabolic enzymes taken from YeastCyc. (B) Evolutionary rate determinants of the 240 proteins in the GO term “signal transduction”. This figure shows that functionally defined protein sets other than TFs have evolutionary rate determinant profiles similar to that of generic proteins. (PDF) [file pcbi.1002734.s004.pdf]
