# Supplementary material for: Regulatory Network Structure as a Dominant Determinant of Transcription Factor Evolutionary Rate
Source: PLoS Comput Biol. 2012 Oct 18;8(10):e1002734. doi: 10.1371/journal.pcbi.1002734 (PMC3475661; doi:10.1371/journal.pcbi.1002734)
Supplement: Table S1 — Spearman correlation coefficients relating TF and target properties in the ChIP-chip network. (DOC) [file pcbi.1002734.s005.doc]

**Supplementary Table S1:** Spearman Correlation Coefficients Relating TF and Target Properties in the ChIP-chip Network

| TF properties  Target properties | TF K_a_/K_s_ | TF Expression | TF PPI degree | TF In-degree |
| --- | --- | --- | --- | --- |
| Fraction of targets in 20% slowest evolving | **-0.23*** | -0.16 | **0.20** | 0.01 |
| Median target K_a_/K_s_ | **0.25*** | **-0.22*** | **-0.24*** | -0.02 |
| Fraction of targets absent in *S. paradoxus* | **0.22** | -0.06 | -0.16 | **0.20** |
| Fraction of targets in 20% most highly expressed | -0.16 | **0.21** | **0.29*** | 0.02 |
| Median target expression | -0.13 | 0.16 | **0.22** | 0.01 |
| Fraction of Targets in 20% most interactive | -0.11 | **0.29*** | **0.22*** | -0.13 |
| Median target PPI degree | -0.15 | **0.18** | **0.19** | -0.10 |

Bold: p-value<0.05

* : p-value<0.01
