# Supplementary material for: Regulatory Network Structure as a Dominant Determinant of Transcription Factor Evolutionary Rate
Source: PLoS Comput Biol. 2012 Oct 18;8(10):e1002734. doi: 10.1371/journal.pcbi.1002734 (PMC3475661; doi:10.1371/journal.pcbi.1002734)
Supplement: Table S2 — Spearman correlation coefficients relating TF and target properties in the network of confirmed edges. (DOC) [file pcbi.1002734.s006.doc]

**Supplementary Table S2:** Spearman Correlation Coefficients Relating TF and Target Properties in the Network of Confirmed Edges

| TF properties  Target properties | TF K_a_/K_s_ | TF Expression | TF PPI degree | TF In-degree |
| --- | --- | --- | --- | --- |
| Targets in 20% slowest evolving^1^ | -0.19 | -0.06 | -0.04 | -0.07 |
| Median target K_a_/K_s_ ^1^ | **0.23** | 0.06 | -0.06 | 0.04 |
| Targets missing in *S. paradoxus* ^2^ | **0.24** | -0.07 | -0.13 | **0.21** |
| Targets in 20% most highly expressed^1^ | -0.16 | 0.13 | **0.23** | 0.06 |
| Median target expression | -0.12 | 0.19 | 0.20 | 0.04 |
| Targets in 20% most interactive^1^ | 0.02 | **0.30*** | **0.27** | -0.07 |
| Median target PPI degree | -0.14 | **0.25** | **0.29*** | -0.03 |

Bold: p-value<0.05

* : p-value<0.01

1 : TFs with 2 or more targets

2 : TFs with 3 or more targets
