# Supplementary material for: Regulatory Network Structure as a Dominant Determinant of Transcription Factor Evolutionary Rate
Source: PLoS Comput Biol. 2012 Oct 18;8(10):e1002734. doi: 10.1371/journal.pcbi.1002734 (PMC3475661; doi:10.1371/journal.pcbi.1002734)
Supplement: Table S3 — Spearman correlation coefficients relating TF and target properties in the network of literature curated edges. (DOC) [file pcbi.1002734.s007.doc]

**Supplementary Table S3:** Spearman Correlation Coefficients Relating TF and Target Properties in the Network of Literature Curated Edges

| TF properties  Target properties | TF K_a_/K_s_ | TF Expression | TF PPI degree | TF In-degree |
| --- | --- | --- | --- | --- |
| Targets in 20% slowest evolving | **-0.22*** | -0.08 | 0.04 | **-0.16** |
| Median target K_a_/K_s_ | **0.26*** | 0.15 | 0.00 | 0.17 |
| Targets missing in *S. paradoxus* | **0.24*** | 0.16 | -0.04 | 0.16 |
| Targets in 20% most highly expressed | **-0.22*** | **0.23*** | **0.21** | -0.14 |
| Median target expression | **-0.27*** | **0.28*** | **0.25*** | -0.05 |
| Targets in 20% most interactive | -0.04 | **0.23*** | **0.28*** | -0.02 |
| Median target PPI degree | **-0.26*** | **0.21** | **0.31*** | -0.06 |

Bold: p-value<0.05

*: p-value<0.01
