# Supplementary material for: Regulatory Network Structure as a Dominant Determinant of Transcription Factor Evolutionary Rate
Source: PLoS Comput Biol. 2012 Oct 18;8(10):e1002734. doi: 10.1371/journal.pcbi.1002734 (PMC3475661; doi:10.1371/journal.pcbi.1002734)
Supplement: Table S4 — Go terms significantly enriched in targets of 75% slowest evolving TFs as compared to the targets of 25% fastest evolving. (DOC) [file pcbi.1002734.s008.doc]

**Supplementary Table S4 :** Go Terms Significantly Enriched in Targets of 75% Slowest Evolving TFs as Compared to the Targets of 25% Fastest Evolving

| GO term | GO ID | Genes | Fold-Enrichment | P-value |
| --- | --- | --- | --- | --- |
| macromolecular complex | GO:0032991 | 1375 | 1.16 | 0.00049 |
| nuclear part | GO:0044428 | 767 | 1.21 | 0.00114 |
| cellular component biogenesis | GO:0044085 | 644 | 1.21 | 0.002342 |
| protein complex | GO:0043234 | 953 | 1.17 | 0.002624 |
| non-membrane-bounded organelle | GO:0043228 | 856 | 1.18 | 0.00298 |
| intracellular non-membrane-bounded organelle | GO:0043232 | 856 | 1.18 | 0.00298 |
| intracellular organelle part | GO:0044446 | 1913 | 1.11 | 0.003372 |
| organelle part | GO:0044422 | 1913 | 1.11 | 0.003372 |
| RNA processing | GO:0006396 | 359 | 1.28 | 0.004795 |
| cellular component organization at cellular level | GO:0071842 | 1028 | 1.14 | 0.007592 |
| cellular macromolecular complex subunit organization | GO:0034621 | 314 | 1.29 | 0.007693 |
| ribonucleoprotein complex | GO:0030529 | 483 | 1.20 | 0.011517 |
| nuclear lumen | GO:0031981 | 521 | 1.19 | 0.013458 |
| ribonucleoprotein complex biogenesis | GO:0022613 | 310 | 1.26 | 0.013639 |
| organelle lumen | GO:0043233 | 690 | 1.16 | 0.013697 |
| intracellular organelle lumen | GO:0070013 | 690 | 1.16 | 0.013697 |
| macromolecular complex subunit organization | GO:0043933 | 335 | 1.25 | 0.014943 |
| cellular component assembly at cellular level | GO:0071844 | 374 | 1.23 | 0.015046 |
| cytoskeleton | GO:0005856 | 167 | 1.39 | 0.015149 |
| ribosome biogenesis | GO:0042254 | 268 | 1.27 | 0.015431 |
| nucleolus | GO:0005730 | 200 | 1.34 | 0.015974 |
| gene expression | GO:0010467 | 976 | 1.13 | 0.017561 |
| organelle organization | GO:0006996 | 849 | 1.14 | 0.01862 |
| cellular component organization or biogenesis at cellular level | GO:0071841 | 1329 | 1.11 | 0.01866 |
| membrane-enclosed lumen | GO:0031974 | 720 | 1.15 | 0.020408 |
| cellular component assembly | GO:0022607 | 393 | 1.21 | 0.021333 |
| cytoskeletal part | GO:0044430 | 155 | 1.37 | 0.027207 |
| cellular macromolecular complex assembly | GO:0034622 | 256 | 1.26 | 0.028279 |
| mitotic cell cycle | GO:0000278 | 218 | 1.29 | 0.029054 |
| mRNA metabolic process | GO:0016071 | 187 | 1.32 | 0.029874 |
| mRNA processing | GO:0006397 | 138 | 1.38 | 0.035091 |
| nuclear envelope | GO:0005635 | 106 | 1.44 | 0.035449 |
| cellular component organization or biogenesis | GO:0071840 | 1403 | 1.09 | 0.036849 |
| modification-dependent protein catabolic process | GO:0019941 | 129 | 1.38 | 0.038839 |
| cellular component biogenesis at cellular level | GO:0071843 | 390 | 1.18 | 0.039871 |
| modification-dependent macromolecule catabolic process | GO:0043632 | 140 | 1.34 | 0.048593 |
| ubiquitin-dependent protein catabolic process | GO:0006511 | 128 | 1.37 | 0.04959 |
