# Supplementary material for: Regulatory Network Structure as a Dominant Determinant of Transcription Factor Evolutionary Rate
Source: PLoS Comput Biol. 2012 Oct 18;8(10):e1002734. doi: 10.1371/journal.pcbi.1002734 (PMC3475661; doi:10.1371/journal.pcbi.1002734)
Supplement: Table S5 — GO terms significantly enriched in target genes of TFs with 10 or more regulators as compared to targets of TFs with 2 or less regulators. (DOC) [file pcbi.1002734.s009.doc]

**Supplementary Table S5:** GO Terms Significantly Enriched in Target Genes of TFs with 10 or more Regulators as Compared to Targets of TFs with 2 or less Regulators

| GO term | GO term ID | # of genes | Fold enrichment | P-value |
| --- | --- | --- | --- | --- |
| Cell periphery | GO:0071944 | 201 | 1.32 | 0.0023 |
| fungal-type cell wall | GO:0009277 | 54 | 1.64 | 0.0042 |
| External encapsulating structure | GO:0030312 | 56 | 1.60 | 0.0052 |
| Cell wall | GO:0005618 | 56 | 1.60 | 0.0052 |
| Oxidoreductase activity | GO:0016491 | 112 | 1.42 | 0.0058 |
| Inorganic cation transmembrane transporter activity | GO:0022890 | 53 | 1.61 | 0.0091 |
| Oxidation reduction process | GO:0055114 | 147 | 1.32 | 0.0096 |
| Plasma membrane | GO:0005886 | 135 | 1.34 | 0.0099 |
| Transporter activity | GO:0005215 | 139 | 1.30 | 0.018 |
| Transmembrane transporter activity | GO:0022857 | 119 | 1.32 | 0.019 |
| Cation transport | GO:0006812 | 69 | 1.44 | 0.022 |
| Ion transport | GO:0006811 | 82 | 1.39 | 0.023 |
| substrate-specific transporter activity | GO:0022892 | 125 | 1.30 | 0.024 |
| Mitochondrial inner membrane | GO:0005743 | 70 | 1.46 | 0.024 |
| substrate-specific transmembrane transporter activity | GO:0022891 | 107 | 1.32 | 0.027 |
| Organelle inner membrane | GO:0019866 | 71 | 1.42 | 0.034 |
| Cation transmembrane transporter activity | GO:0008324 | 54 | 1.46 | 0.035 |
| Ion transmembrane transport | GO:0034220 | 67 | 1.40 | 0.036 |
| Homeostatic process | GO:0042592 | 74 | 1.40 | 0.038 |
| Ion transmembrane transporter activity | GO:0015075 | 62 | 1.41 | 0.042 |
