# Supplementary material for: Regulatory Network Structure as a Dominant Determinant of Transcription Factor Evolutionary Rate
Source: PLoS Comput Biol. 2012 Oct 18;8(10):e1002734. doi: 10.1371/journal.pcbi.1002734 (PMC3475661; doi:10.1371/journal.pcbi.1002734)
Supplement: Table S6 — GO terms significantly enriched in target genes of TFs with 2 or less regulators as compared to targets of TFs with 10 or more regulators. (DOC) [file pcbi.1002734.s010.doc]

**Supplementary Table S6:** GO Terms Significantly Enriched in Target Genes of TFs with 2 or less Regulators as Compared to Targets of TFs with 10 or more Regulators

| GO term | GO term ID | # of genes | Fold enrichment | P-value |
| --- | --- | --- | --- | --- |
| intracellular | GO:0005622 | 2337 | 1.07 | 0.0022 |
| non-membrane-bounded organelle | GO:0043228 | 595 | 1.28 | 0.0032 |
| intracellular non-membrane-bounded organelle | GO:0043232 | 595 | 1.28 | 0.0032 |
| membrane-enclosed lumen | GO:0031974 | 502 | 1.30 | 0.0041 |
| intracellular part | GO:0044424 | 2323 | 1.06 | 0.0047 |
| intracellular organelle lumen | GO:0070013 | 483 | 1.30 | 0.0049 |
| organelle lumen | GO:0043233 | 483 | 1.30 | 0.0049 |
| organelle organization | GO:0006996 | 594 | 1.26 | 0.0054 |
| cellular metabolic process | GO:0044237 | 1716 | 1.10 | 0.0069 |
| transferase activity | GO:0016740 | 384 | 1.32 | 0.0077 |
| cellular component organization or biogenesis at cellular level | GO:0071841 | 915 | 1.18 | 0.0084 |
| cellular protein metabolic process | GO:0044267 | 606 | 1.24 | 0.0092 |
| Cellular macromolecule metabolic process | GO:0044260 | 1168 | 1.14 | 0.0109 |
| intracellular organelle part | GO:0044446 | 1318 | 1.12 | 0.0115 |
| organelle part | GO:0044422 | 1318 | 1.12 | 0.0115 |
| protein metabolic process | GO:0019538 | 650 | 1.22 | 0.0122 |
| primary metabolic process | GO:0044238 | 1623 | 1.10 | 0.0140 |
| macromolecule metabolic process | GO:0043170 | 1207 | 1.13 | 0.0144 |
| cellular process | GO:0009987 | 2329 | 1.05 | 0.0148 |
| nuclear lumen | GO:0031981 | 359 | 1.30 | 0.0157 |
| nuclear part | GO:0044428 | 511 | 1.24 | 0.0180 |
| cellular component organization or biogenesis | GO:0071840 | 964 | 1.15 | 0.0195 |
| ribonucleoprotein complex | GO:0030529 | 348 | 1.28 | 0.0220 |
| cellular component organization at cellular level | GO:0071842 | 704 | 1.18 | 0.0229 |
| organelle | GO:0043226 | 2014 | 1.07 | 0.0237 |
| intracellular organelle | GO:0043229 | 2014 | 1.07 | 0.0237 |
| macromolecular complex | GO:0032991 | 950 | 1.14 | 0.0265 |
| ribosomal small subunit biogenesis | GO:0042274 | 384 | 1.83 | 0.0267 |
| purine nucleotide binding | GO:0017076 | 214 | 1.25 | 0.0318 |
| translation | GO:0006412 | 380 | 1.34 | 0.0360 |
| purine ribonucleoside triphosphate binding | GO:0035639 | 381 | 1.23 | 0.0427 |
| purine ribonucleotide binding | GO:0032555 | 381 | 1.23 | 0.0428 |
| ribonucleotide binding | GO:0032553 | 2581 | 1.23 | 0.0428 |
| cell | GO:0005623 | 2581 | 1.03 | 0.0437 |
| cell part | GO:0044464 | 170 | 1.03 | 0.0437 |
| vesicle-mediated transport | GO:0016192 | 119 | 1.39 | 0.0448 |
| cellular lipid metabolic process | GO:0044255 | 1859 | 1.50 | 0.0464 |
| cytoplasm | GO:0005737 | 2337 | 1.06 | 0.0496 |
